# Supplementary material for: Plasmids, prophages, and defense systems are depleted from plant microbiota genomes
Source: Genome Biol. 2025 Jun 11;26:163. doi: 10.1186/s13059-025-03641-3 (PMC12153167; doi:10.1186/s13059-025-03641-3)
Supplement: Supplementary file 4 — Additional file 4: Fig. S1: Distribution of intact defense systems in PA and NPA bacteria Contains two panels: (a) heatmap showing significant enrichment or depletion of defense systems across 19 bacterial families comparing PA vs. NPA genomes; (b) bar plot of the 10 most abundant defense systems per family. [file 13059_2025_3641_MOESM4_ESM.docx]

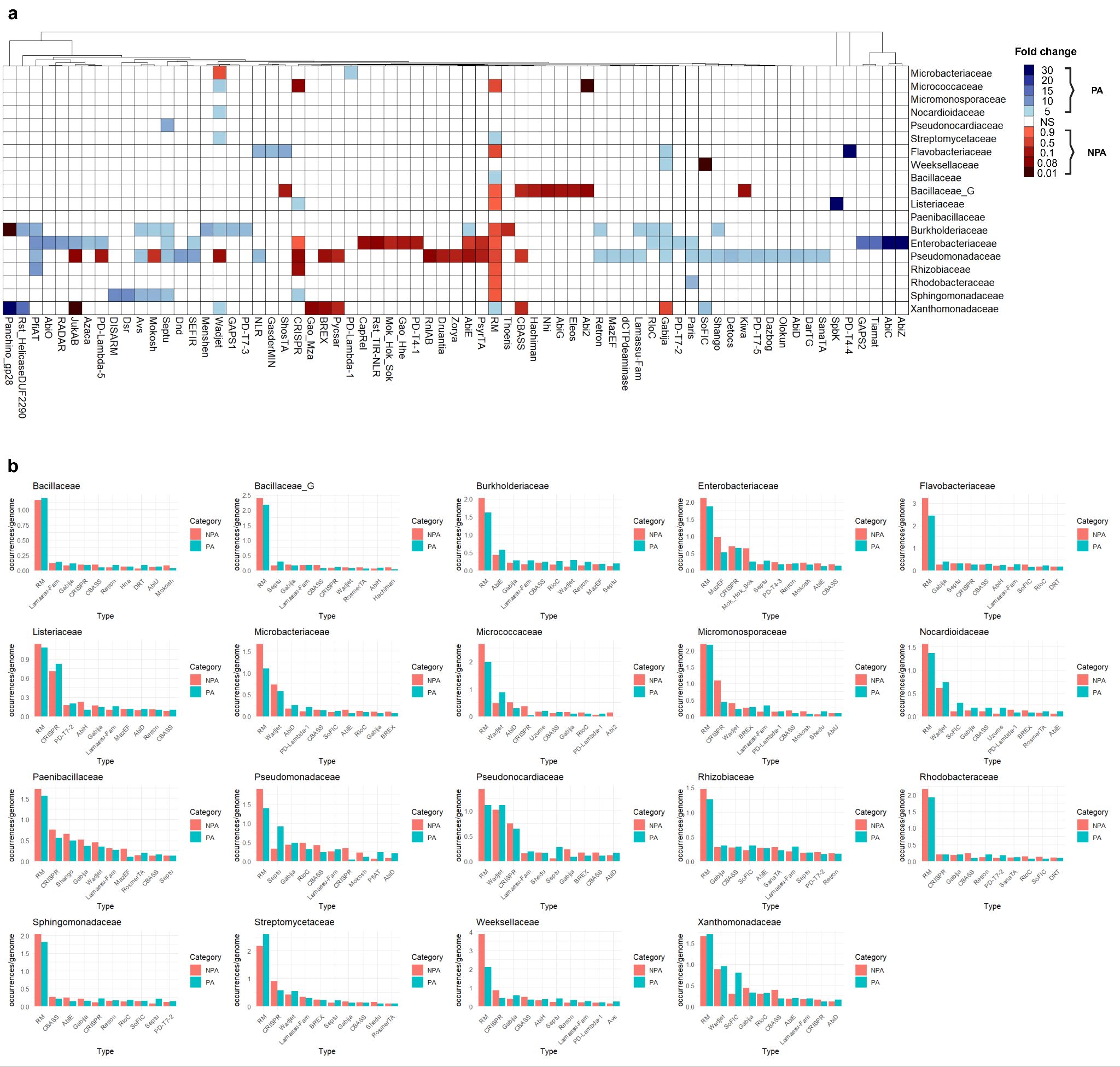


**Supplementary Figure 1. Distribution of all intact defense systems in PA and NPA bacteria across families. a.** A heatmap showing the significant defense systems that are enriched (blue) or depleted (red) in PA bacteria. Values are the mean fold change per genomes of each family. Each square shows PA/NPA fold change if there is a significant difference in the abundance of the defense system between the genomes of PA vs. NPA bacteria from a given family (Wilcoxon test following FDR correction). **b.** Number of occurrences/genome of the ten most abundant defense systems per 19 families analyzed.
